# Characterization and Quenching of Autofluorescence in Piglet Testis Tissue and Cells

**DOI:** 10.1155/2012/820120

**Published:** 2012-08-30

**Authors:** Yanfei Yang, Ali Honaramooz

**Affiliations:** Department of Veterinary Biomedical Sciences, Western College of Veterinary Medicine, University of Saskatchewan, 52 Campus Drive, Saskatoon, SK, Canada S7N 5B4

## Abstract

Significant intrinsic fluorescence in tissues and in disassociated cells can interfere with fluorescence identification of target cells. The objectives of the present study were (1) to examine an intrinsic fluorescence we observed in both the piglet testis tissue and cells and (2) to test an effective method to block the autofluorescence. We observed that a number of granules within the testis interstitial cells were inherently fluorescent, detectable using epifluorescence microscopy, confocal laser scanning microscopy, and flow cytometry. The emission wavelength of the autofluorescent substance ranged from 425 to 700 nm, a range sufficiently broad that could potentially interfere with fluorescence techniques. When we treated the samples with Sudan Black B for different incubation times, the intrinsic fluorescence was completely masked after treatment for 10–15 min of the testis tissue sections or for 8 min of the testis cells, without compromising specific fluorescence labeling of gonocytes with lectin *Dolichos biflorus* agglutinin (DBA). We speculate that the lipofuscin or lipofuscin-like pigments within Leydig cell granules were mainly responsible for the observed intrinsic fluorescence in piglet testes. The method described in the present study can facilitate the identification and characterization of piglet gonocytes using fluorescence microscopy.

## 1. Introduction

The mammalian testis is composed of seminiferous tubules, primarily containing germ and Sertoli cells, and interstitial tissues containing Leydig cells. As the earliest identifiable germ cell progenitors, primordial germ cells (PGCs) proliferate and differentiate in the fetal testis gonad into gonocytes [1–3]. After birth, gonocytes proliferate in the testis and develop into spermatogonial stem cells (SSCs) prior to puberty [4, 5]. In the mature testis, SSCs initiate and maintain the continuity of spermatogenesis through self-renewal, proliferation, and differentiation to produce daughter germ cells eventually leading up to spermatozoa [4, 6]. In the neonatal testis, gonocytes are the only germ cells present [7–11], and although they give rise to SSCs and are considered germline stem cells, there is controversy as to whether gonocytes have the capability to initiate spermatogenesis on their own, that is, without first developing into SSCs [12–16]. Compared with PGCs and SSCs, gonocytes are the least investigated germline progenitor cells [17]; therefore, obtaining new knowledge about gonocytes may also shed light on the germline stem cells as a whole.

 Although gonocytes can be identified *in situ* and in histological cross-sections by their distinctive topography within the seminiferous cords/tubules and unique morphological attributes [12, 18], specific biomarkers are required for their accurate quantification. The unique expression of biomarkers in/on gonocytes may also indicate specific cellular functions or uncover important biological information about them. 

Fluorescence labeling is commonly and widely applied in biomedical investigations and in laboratory diagnosis to locate specific antigens/biomarkers on/in cells and tissues. Fluorophore-conjugated antibodies enable the quantitative detection of the target antigens, often using multiple fluorescent probes simultaneously [19, 20]. When multiple fluorescent probes are applied, attention should be given to the interference among fluorophores, especially to the autofluorescence (intrinsic fluorescence) present in certain cells or tissues which could interfere with the fluorescence signal of interest by creating false-positive results. 

While examining piglet gonocytes, we encountered an intense autofluorescence in both neonatal testis tissue and disassociated testis cells. Although expected in mature animals, this intrinsic fluorescence has not been reported in neonatal piglet testes and could blur the distinction between specific and nonspecific fluorescence signals, interfering with characterization of piglet gonocytes among dissociated testis cells. Therefore, in the present study, we examined the intrinsic fluorescence in piglet testis cells and evaluated a method to mask such autofluorescence.

## 2. Materials and Methods

### 2.1. Testes Collection and Tissue Preparation

Testes were collected after castration of one-week-old Yorkshire cross-piglets (*n* = 32, Camborough-22 × Line 65, PIC Canada Ltd., Winnipeg, MB, Canada) in a university-affiliated swine facility. For comparison, testes from two-month-old and mature (24 ± 6 months) pigs from the same farm were also collected (*n* = 3 each). Testes were then transferred on ice to the laboratory in Dulbecco's phosphate-buffered saline (DPBS, cat. no. 20-031-CV, Mediatech, Manassas, VA, USA) within 2 h after collection. On arrival, the testes were rinsed three times with DPBS, the tunica albuginea, rete testis, and excessive connective tissue were then removed. Experimental procedures involving animals were approved by the University of Saskatchewan's Animal Research Ethics Board and adhered to the Canadian Council on Animal Care guidelines for humane animal use.

### 2.2. *In Situ* Detection of Autofluorescence

Small fragments of freshly collected pig testis tissues in DPBS were gently disassociated into seminiferous cords using fine needles in culture dishes and examined for autofluorescence with both epifluorescent (Leica DMI 6000B equipped with A (ultraviolet), I3 (green), and N21 (red) filter cubes) and confocal laser scanning microscopes (Leica TCS SP5, Leica Microsystems, Mannheim, Germany) with a 20x objective and an excitation laser of 405 nm, and acquisition of signals from spectrums of 440–490 nm (blue), 495–570 nm (green), 575–620 nm (yellow), and 625–780 nm (red). 

Testis tissue samples were also fixed in Bouin's solution, paraffin embedded and processed to prepare tissue sections using standard histological procedures. After deparaffinisation, rehydration, and hematoxylin, and eosin staining, slides were examined under both epifluorescent and confocal laser scanning microscopes.

### 2.3. Examination of Autofluorescence in Isolated Piglet Testis Cells

Testis cells were collected using a two-step digestion method with minor modifications [21, 22]. Briefly, testis tissue pieces of approximately 100 mg were minced with fine scissors and digested with 0.2% w/v collagenase IV (cat. no. C-5138, Sigma-Aldrich, Oakville, ON, Canada), 0.1% w/v hyaluronidase (cat. no. H-3884, Sigma-Aldrich), and 0.01% w/v DNase I (cat. no. DN25, Sigma-Aldrich) in Dulbecco's modified Eagle's medium (DMEM, cat. no. 10-013-CM, Mediatech) at 37°C for 15 min with agitation every 5 min. After centrifugation at 15 g at 16°C for 1 min and discarding the supernatant, tissue pellets were further digested with 0.25% w/v trypsin with 2.21 mM EDTA (cat. no. 25-053-CI, Mediatech) at 37°C for 5 min. Undiluted fetal bovine serum (FBS, cat. no. A15-701, PAA Laboratories GmbH, Etobicoke, ON, Canada) was added to stop the digestion, and the cell suspension triturated with a 1 ml pipette tip before filtration through a 40 *μ*m filter (cat. no. 352340, BD Biosciences, Mississauga, ON, Canada). Erythrocytes were then removed with a lysis buffer containing NH_4_CL 156 mM, KHCO_3_ 10 mM, Na_2_EDTA 0.1 mM [23, 24] at a ratio of 4 : 1 (buffer : cell suspension) for 15 min at room temperature. After centrifugation at 600 g for 4 min and rinsing with 10 mL of 10% FBS-DMEM, newly disassociated cells were suspended in DPBS and smeared onto poly-D-lysine treated slides. A subset of slides were examined immediately using both epifluorescent and laser scanning microscopes, while the remaining slides were dried in air and stored at −20°C for autofluorescence blocking and DBA staining. Flow cytometry (Partec CyFlow Space, Partec GmbH, Münster, Germany) was also used to characterize the autofluorescence of the freshly isolated testis cells after staining with 4,6-diamino-2-phenyl indole (DAPI, cat. no. D-9542, Sigma-Aldrich) for 3 min. To probe the autofluorescence spectrum, serial excitation lasers (with wavelengths of 405, 458, 476, 488, 514, 543, or 633 nm) with emission wavelengths ranging from 415 to 800 nm were applied to the freshly isolated testis cells using a confocal laser scanning microscope. The autofluorescence intensity following different excitation and emission wavelengths was then subjectively evaluated. 

### 2.4. Duration of Autofluorescence in Cultured Testis Cells

Freshly isolated testis cells were cultured in 6-well plates with cover slides at the bottom of plates, in DMEM containing 10% FBS at 37°C in a 5% CO_2_ humidified atmosphere for 6 days. Every 24 hours, during the culture, cover slides (*n* = 3/day) with cells on top were collected, rinsed with DPBS, and autofluorescence detected using a laser scanning microscope using excitation with a 405 nm laser line.

### 2.5. Elimination of the Autofluorescence for Identification of Gonocytes *In Situ*

After deparaffinisation and rehydration, the processed testis tissue sections were rinsed with DPBS and incubated with 5% w/v bovine serum albumin (BSA, cat. no. A7638, Sigma-Aldrich) in DPBS at 37°C for 30 min in humidified atmosphere and stained overnight with a fluorescein-conjugated lectin *Dolichos biflorus* agglutinin (DBA) [11] (1 : 100, cat. no. FL-1031, Vector Labs, Burlington, ON, Canada) in humidified atmosphere. After rinsing with DPBS, the sections were incubated with 0.3% w/v Sudan Black B (SBB, cat. no. 3545-12, EMD Chemicals, Gibbstown, NJ, USA) in 70% ethanol at 37°C for 0, 3, 5, 8, 10, 12, 15, or 20 min in humidified atmosphere, rinsed with DPBS, and stained with DAPI for 3 min. The sections were then mounted (cat. no. H-1000, Vector Labs) and examined by a laser scanning confocal microscope for sequential scanning and detection of DAPI and fluorescein, respectively, followed by merging and saving of the images. We chose to test SBB because previously it was found to be the most effective dye in reducing lipofuscin-like autofluorescence in different tissues from multiple species [25, 26].

### 2.6. Elimination of the Autofluorescence for Identification of Gonocytes *In Vitro*

After thawing at room temperature, cell smears were fixed in Bouin's solution for 2-3 min, rinsed in DPBS, blocked using 5% BSA for 15 min at 37°C in humidified atmosphere, rinsed again with DPBS, and incubated with a fluorescein-labeled DBA overnight (1 : 100) in humidified atmosphere. After rinsing with DPBS and incubation with 0.3% SBB in 70% ethanol for 0, 3, 5, 8, 10, 12, 15, or 20 min, cell smears were rinsed in DPBS, stained with DAPI for 2 min, mounted and observed under a laser scanning confocal microscope. DAPI and fluorescein were sequentially scanned using the laser scanning microscope using excitation with a 405 nm laser.

## 3. Results

### 3.1. *In Situ* Autofluorescence

**Figure 1:**
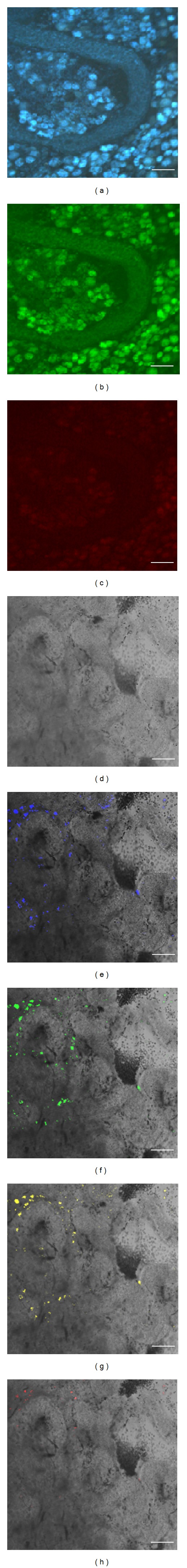
Autofluorescence observed in whole-mount seminiferous cords by epifluorescent and confocal laser scanning microscopes. Seminiferous cords were dissociated from 1-wk-old piglet testes and examined under an epifluorescent microscope (a–c) equipped with filters of A (a), I3 (b), and N21 (c). Confocal laser scanning microscopy with excitation laser of 405 nm, brightfield (d), or brightfield overlaid with acquired signal from spectrums of blue (440–490 nm, e), green (495–570 nm, f), yellow (575–620 nm, g), and red (625–780 nm, h). Scale bars, 100 *μ*m.

**Figure 2:**
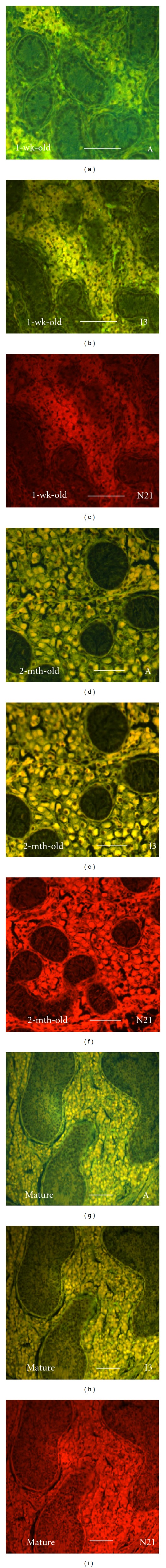
Autofluorescence in testis tissue sections from pigs of different ages examined using an epifluorescent microscope. Testis tissues from 1-wk-old (a–c), 2-month-old (d–f), and mature (g–i) pigs were fixed, sectioned, and examined for intrinsic fluorescence using an epifluorescent microscope equipped with filters of A (a, d, g), I3 (b, e, h), and N21 (c, f, i). Scale bars, 100 *μ*m.

**Figure 3:**
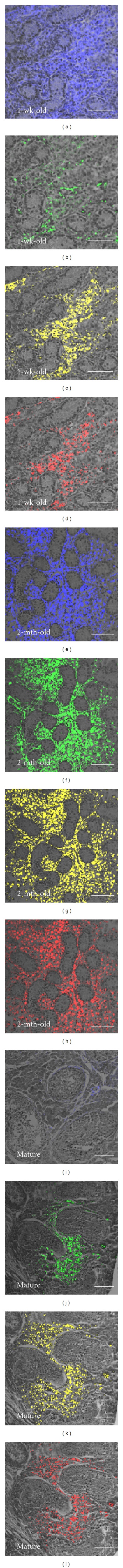
Autofluorescence in testis tissue sections from pigs of different ages observed using a confocal laser scanning microscope. Testis tissues from 1-wk-old (a–d), 2-month-old (e–h), and mature (i–l) pigs were fixed, sectioned, and examined for intrinsic fluorescence under a confocal laser scanning microscope with excitation by a 405 nm laser and acquisition of signal from spectrums of blue (440–490 nm; a, e, i), green (495–570 nm; b, f, g), yellow (575–620 nm; c, g, k), and red (625–780 nm; d, h, l) with brightfield overlay. Scale bars, 100 *μ*m.

An intense fluorescence was consistently detected by both epifluorescence and confocal laser scanning microscopes in testes from neonatal, prepubertal, and mature pigs (Figures 1, 2, and 3). The autofluorescence was exclusively observed in the testis interstitial cells *in situ, *mainly in the cytoplasm of most Leydig cells that contained strongly fluorescent intracellular granules, although some Leydig cells did not seem to contain the autofluorescent granules (Figures 1–3).

### 3.2. Autofluorescence in Disassociated Testis Cells

**Figure 4:**
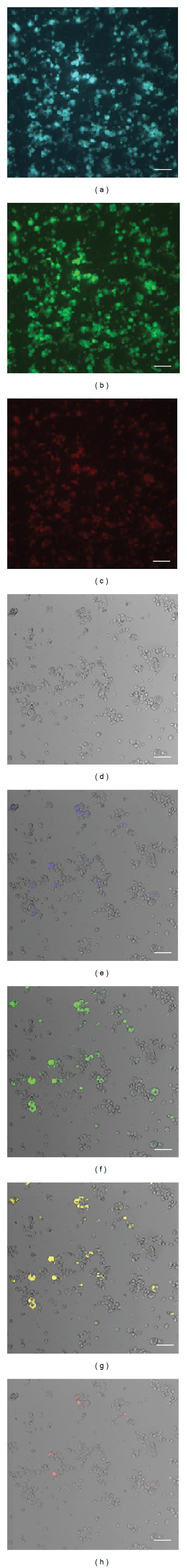
Autofluorescence detected in freshly isolated testis cells using epifluorescent and confocal laser scanning microscopes. Testis cells were isolated from 1-wk-old piglet testes and examined under an epifluorescent microscope (a–c) equipped with filters of A (a), I3 (b), and N21 (c). Confocal laser scanning microscopy using an excitation laser of 405 nm, brightfield (d), or brightfield overlaid with acquired signals from spectrums of blue (440–490 nm, e), green (495–570 nm, f), yellow (575–620 nm, g), and red (625–780 nm, h). Scale bars, 50 *μ*m.

**Figure 5:**
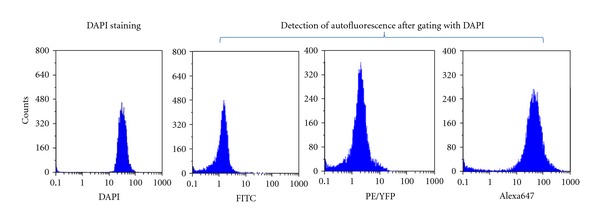
Flow cytometry analysis of autofluorescence in freshly isolated testis cells. Testis cells were isolated from 1-wk-old piglet testes, stained with DAPI, and assayed with a flow cytometer equipped with detectors for FITC, PE/YFP, and Alexa647 with DAPI gating to specifically detect autofluorescence from piglet testis cells.

When freshly isolated testis cells were observed using fluorescent or confocal laser scanning microscope, a strong autofluorescence was detected in the cytoplasm of some small and large round cells (~10 *μ*m versus ~20 *μ*m), but the fluorescence intensity differed among the observed autofluorescent testis cells (Figure 4). The intrinsic fluorescence signal was also detectable in testis cells using flow cytometry, with emission wavelengths overlapping those of fluorophores such as FITC, PE, and Alexa 647 (Figure 5). 

### 3.3. Emission Wavelength of the Autofluorescence

**Figure 6:**
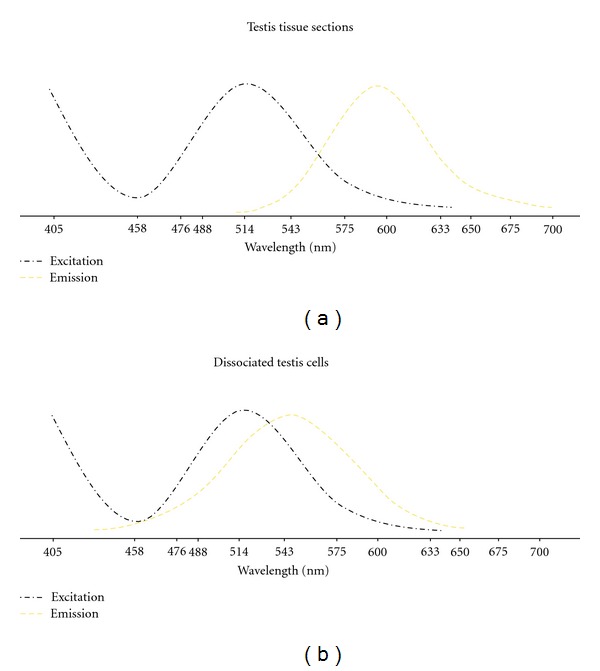
Autofluorescence spectrum assessed using a confocal laser scanning microscope. Piglet testis tissue sections and dissociated cells were excited with lasers of 405, 458, 476, 488, 514, 543, and 633 nm. Emission wavelengths excited at 405 nm were probed every 10 nm ranging from 410 to 750 nm, and the signal intensity was subjectively evaluated. Intensity of the signal is shown as tendency and may not demonstrate the actual signal strength.

Excitation lasers of different wavelengths affected the autofluorescence emission wavelengths of the freshly isolated interstitial cells. *In situ* autofluorescence was detected with emission wavelengths ranging from 425 to 700 nm with strong signals between 480 and 620 nm, spanning the spectrums of green, yellow, and red, thereby interfering with the most commonly used fluorophores (Figure 6).

### 3.4. Autofluorescence in Cultured Testis Cells

**Figure 7:**
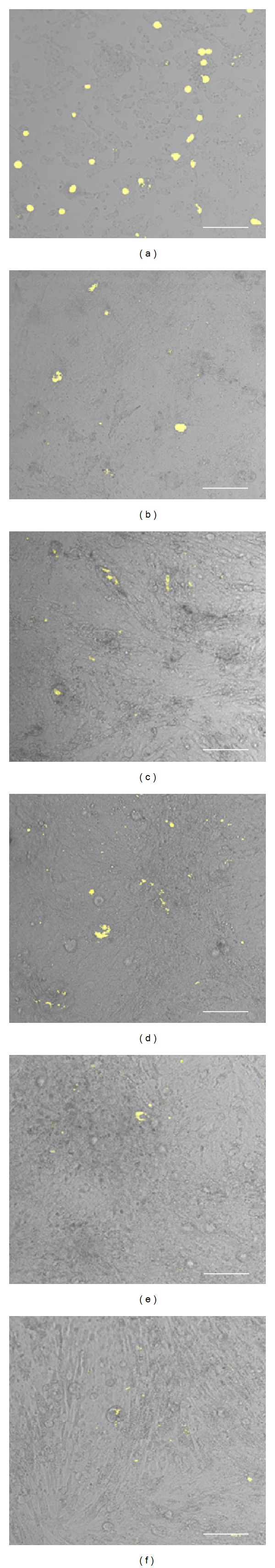
Autofluorescence in cultured testis cells. One-wk-old piglet testis cells were cultured *in vitro* for 6 days and examined for autofluorescence using a confocal laser scanning microscope and excited with a 405 nm laser and detection of emissions within 575–620 nm (yellow with brightfield overlay, (a–f) corresponding to 1–6 days following culture, resp.). Scale bars, 100 *μ*m.

To determine the fate of the autofluorescence *in vitro*, testis cells were cultured for 6 days and examined daily with epifluorescent and confocal laser scanning microscopes. Although the autofluorescence was consistently observed for at least 6 days, the extent and intensity tended to decrease over time (Figure 7). 

### 3.5. Quenching of the Autofluorescence with Sudan Black B for Gonocyte Identification

**Figure 8:**
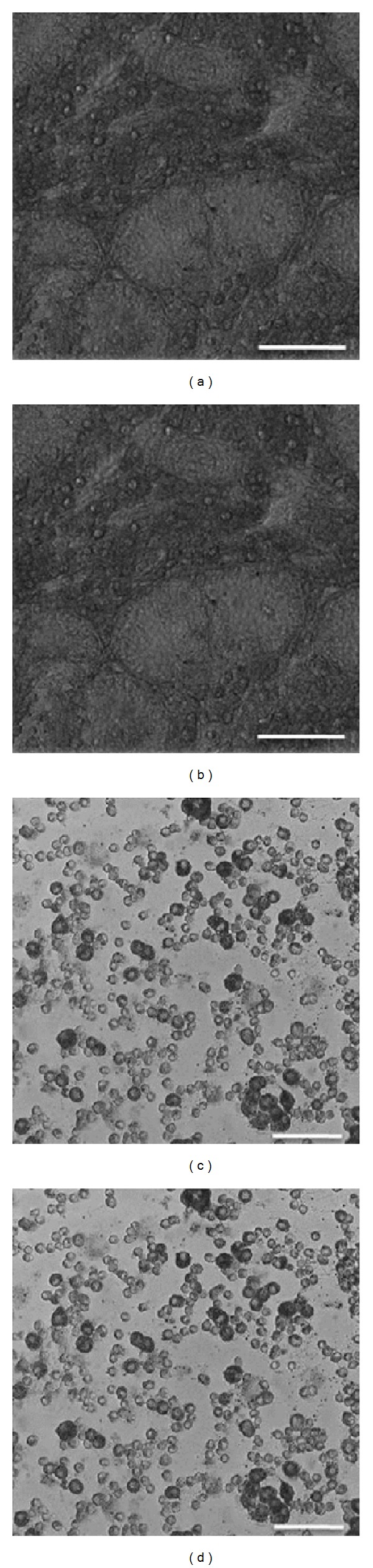
Autofluorescence blocked with Sudan Black B staining of testis cells *in situ* and *in vitro*. Piglet testis tissue sections (a, b) and dissociated cells (c, d) were stained with Sudan Black B, examined under a brightfield microscope (a, c) and a confocal laser scanning microscope, excited with a 405 nm laser with detection of emissions within 575–620 nm (b, d) (with brightfield overlay). Scale bars, 100 *μ*m.

**Figure 9:**
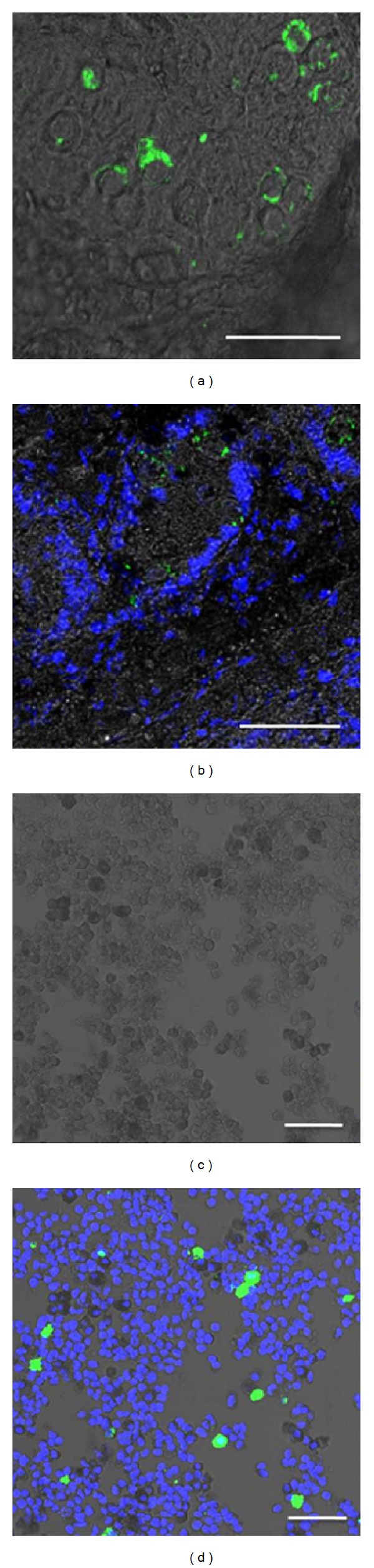
Identification of gonocytes with DBA staining, following the masking of autofluorescence by Sudan Black B *in situ* and *in vitro*. Piglet testis tissue sections (a, b) and dissociated cells (c, d) were stained with FITC-labeled lectin DBA and DAPI, followed by Sudan Black B staining and imaging with a confocal laser scanning microscope with brightfield overlay. Scale bars, 100 *μ*m.

When SBB was applied to testis cells *in situ* and *in vitro* for different incubation times, the expressed autofluorescence was completely blocked after staining for approximately 12 or 8 min, respectively (Figure 8). Compared with nontreated testis cells, SBB completely quenched the autofluorescence in testis cells both *in situ* and *in vitro*, while not blocking the specific staining of gonocytes with DBA (Figure 9).

## 4. Discussion

Autofluorescence within the target tissue or cells could interfere with detection of specific signals from the labeling fluorophores, leading to inaccurate or even false-positive results. In preliminary observations, we noticed that because of the strong innate autofluorescence in isolated testis cells, identifying piglet gonocytes using fluorescence staining was difficult. In the present study, we observed that the autofluorescence was limited to the interstitial tissue/cells of the testis (Figures 1–3), and the source was primarily the intrinsically fluorescent granules within the cytoplasm of Leydig cells. Gonocytes did not emit fluorescence. The autofluorescence had a wide excitation and emission spectrum, strong enough to potentially mimic the appearance of fluorescence labeling. When testis cells were cultured, this intrinsic fluorescence decreased in intensity (Figures 6 and 7). Treatment of the testis tissue and cells with the lysochrome SBB completely masked the intrinsic fluorescence while not compromising with the identification of gonocytes through detection of specific fluorescent signal (Figures 8 and 9).

The sources of intrinsic fluorescence in tissue and cell samples can be divided into natural and fixative-induced fluorescence causes. In the present study, the autofluorescence was equally detectable in the testis tissue in both fresh and fixed samples (Figures 1–4). This may imply that the observed autofluorescence was indeed intrinsic to the cells and not acquired during the processing. Flavins and porphyrins are among the most common natural fluorescence substances in fresh tissues/cells, but they are generally extracted during fixation and dehydration processes. Natural sources of the remaining autofluorescence in fixed and processed tissues include elastin, collagen, and lipofuscin. While elastin and collagens comprise major sources of natural autofluorescence in the extracellular matrices, lipofuscin is the main intracellular natural source of autofluorescence in both fresh and fixed samples [27–29]. Throughout our observations, the intrinsic fluorescence was consistently detected within interstitial cells in both *in vitro* and *in situ* (i.e., in freshly disassociated testis cells, tissue whole mounts, tissue sections, and testis cell culture), indicating that the source of autofluorescence in the neonatal piglet testes may likely be lipofuscin or lipofuscin-like pigments. 

Lipofuscin is a nondegradable autofluorescent pigment (composed mostly of lipid and protein) which could not be exported from the cells, therefore, accumulates in an almost linear manner with increasing age in the cells, and is stored in the lysosomes as waste materials [30, 31]. In addition to aging, stress has also been cited as a potential reason for accumulation of lipofuscin. In mice under chronic stress treatment (noise exposure), similar granules were reported to form and accumulate particularly in Leydig cells, and lipofuscin was suggested as the fluorescent substance in the granules [32]. While aging or chronic stress is unlikely causes of lipofuscin accumulation in the cells of newborn animals, perhaps other factors may also regulate the accumulation of lipofuscin or lipofuscin-like pigments in the neonatal testes. An intrinsic fluorescence has also been reported in neonatal mouse testis cells, with intensities comparable to the labeling fluorophores [15, 33] and in neonatal bovine testis cells, interfering with the purification of spermatogonia using fluorescence-activated cell sorting [34]. Fluorescent granules were also observed in Leydig cells in testes of mature crossbred boars [35]; however, to our knowledge autofluorescence has not been reported in neonatal piglet testes. It is important to note that autofluorescence with broad excitation and emission wavelengths is considered typical of lipofuscin and lipofuscin-like pigments [26], and quenching of such autofluorescence by lipid staining (e.g., SBB) was suggested to further indicate the lipofuscin origin of the autofluorescence [26, 30, 31, 36]. Therefore, the fact that the autofluorescence in the present study had a broad spectrum (425 to 700 nm with solid signal from 480 to 620 nm and was observable with widely used filters) and was quenchable with SBB supports the speculation that lipofuscin or lipofuscin-like pigments are mainly responsible for the autofluorescence in the piglet testes. 

Staining of different neural tissues from multiple species with several dyes leads to the conclusion that SBB was the most in reducing the lipofuscin-like autofluorescence, without causing the loss of the specific fluorescence signal in immune staining [25, 26]. In the present study, SBB also completely masked the intrinsic fluorescence of pig testis cells both *in situ* and *in vitro* but allowed the identification of gonocytes with fluorescein-labeled DBA. While degradation and exocytosis do not remove lipofuscin from the cells, mitotic division is the only reported mechanism that can reduce the lipofuscin concentration within cells [30, 31, 37]. In the present study, decreasing autofluorescence was observed over the duration of the testis cells culture. We speculate that the lipofuscin content of the cells was divided into the newly formed cells. 

## 5. Conclusions

In conclusion, we characterized an intrinsic fluorescence in piglet testes and showed that the use of SBB can completely quench this autofluorescence, without interfering with identification of specific testis cells by fluorescence microscopy.
